# Employing the London Atlas in the Age Estimation of a Select South African Population

**DOI:** 10.3390/dj10090171

**Published:** 2022-09-09

**Authors:** Sundika Ishwarkumar, Pamela Pillay, Manogari Chetty, Kapil Sewsaran Satyapal

**Affiliations:** 1 Department of Human Anatomy and Physiology, Faculty of Health Sciences, University of Johannesburg, Doornfontein Campus, P.O. Box 524, Auckland Park, Johannesburg 2006, South Africa; 2 Department of Clinical Anatomy, School of Laboratory Medicine and Medical Sciences, College of Health Sciences, University of KwaZulu-Natal, Westville Campus, Private Bag X54001, Durban 4000, South Africa; 3 Department of Craniofacial Biology, Faculty of Dentistry, University of Western Cape, Cape Town 7535, South Africa

**Keywords:** age estimation, forensic odontology, London Atlas, panoramic radiographs, population-specific data, radiographic analysis

## Abstract

Dental age estimation in the living and deceased is a fundamental aspect of forensic sciences, civil cases, medico-legal proceedings and clinical dentistry. Accordingly, this study aimed to validate the accuracy and reproducibility of the London Atlas in a select South African sample of KwaZulu-Natal. In this cross-sectional study, 760 digital panoramic radiographs (*n* = 760) aged between 5.00 and 23.99 years were retrospectively reviewed through consecutive sampling. Each radiograph was assessed and assigned a dental age in accordance with the London Atlas of Human Tooth Development and Eruption by AlQahtani et al. (2010). The London Atlas overestimated age with a mean difference of −0.85 to −1.26 years in the selected South African sample of KwaZulu-Natal. A statistically significant difference between the chronological and estimated dental ages was recorded. Furthermore, the South African Black and Indian males had a higher overestimation of age than their female counterparts, with a mean difference of 0.13 and 0.07 years, respectively. This overestimation was less in the South African Indian population in comparison to the SA Black population. This outcome resulted in the creation of the KZN population- and sex-specific charts and atlases for the two selected cohorts of KwaZulu-Natal. The KZN Atlases were found to be more accurate in the selected sample, with a mean absolute error of 0.57 years and no statistically significant differences between the chronological and estimated dental ages.

## 1. Introduction

The estimation of age in the living has become a fundamental aspect of forensic sciences and medico-legal situations involving unidentified individuals awaiting adoption, asylum applications, civil issues and judicial procedures [1,2]. It is also used to determine if an individual is of legal age for criminal responsibility [1,2]. Moreover, age estimation is important for the identification of unknown remains, particularly in crime scenes and mass or natural disasters [1,2]. Clinically, age estimation is required for orthodontic, pediatric and endocrinology treatment planning [1,3]. 

Over the last few decades, forensic dentistry has gained significant “popularity” in the field of forensic science, as dental development is primarily controlled by genes and is less affected by extrinsic factors (i.e., nutrition and environmental factors) in comparison to skeletal development [3,4]. According to literature reports, the assessment of dental development using panoramic radiographs is an exceedingly reliable and precise method for dental age estimation [3,4]. In fact, this has emerged as the preferred technique for dental age estimation due to it cost-effectiveness, ease of use and low radiation exposure. 

Similarly, dental atlases are considered to be a useful tool in estimating dental age in mortuaries and in the event of mass disasters by narrowing the search for unidentified individuals, as this method is simple, easy and quick to perform relative to other methods [3,4,5,6]. In particular, AlQahtani et al. [7] established the London Atlas of Human Tooth Development and Eruption in 2010. This atlas consists of thirty-one age categories (28 weeks in-utero to 23 years) based on dental development and eruption sequences. The tooth development and alveolar eruption stages were identified in accordance with the modified Moorrees stages [8,9]. Sharma and Wadhwan [3] and Koc et al. [4] concluded that the London Atlas is advantageous, as it is more user friendly due to its simplicity and convenience. However, Alkandiri et al. [6] and Sezer and Carikcioglu [10] reported that the London Atlas was less accurate in their samples than the Simple Average and Haavikko methods in the Kuwaiti and Turkish populations, respectively. Moreover, Carl [11] noted that the Willems method outperformed the London Atlas in their doctoral thesis conducted on a German population.

Alshihri et al. [12] recommended that the universal applicability of the London Atlas must be verified in different population groups to prove its practicality and validity. Currently, it is used for age estimation in Western population groups only, viz., Portugal, the Netherlands, the United States, Canada, France, and the United Kingdom [7,8]. However, there is paucity of literature regarding the applicability of the London Atlas to other population groups, e.g., Sub-Saharan Africa. In 2018, Esan and Schepartz [13] compared the London Atlas with the WITS Atlas, which the aforementioned authors created using the Demirjian method for the SA Black population of Gauteng province in South Africa. Unfortunately, the comparison revealed that the London Atlas was not suitable for age estimation in this population group [13]. The study by Esan and Schepartz [13] compared the developmental stages of the London Atlas to the WITS Atlas; however, they did not directly investigate the applicability of the London Atlas to the South African (SA) population. It should be noted that dissimilarities between the London and WITS Atlases may be attributed to the established methodology: the London Atlas is based on the Moorrees Classification Scheme, with a median value used to formulate the atlas, while the WITS Atlas utilized the Demirjian method and was formulated using the modal values of their sample [2]. Moreover, uneven sample distribution was noted for the sex and age groups in the sample of the WITS Atlas, while the London Atlas sampled an equal distribution in their study [8,13]. Furthermore, Esan and Schepartz conducted a community-based study in the Gauteng region of South Africa, and literary reports documented variation among local sub-population groups and regional differences [12,14,15,16,17]. These differences should be taken into consideration, as the best estimates for age estimation are obtained from local population-specific standards [14,15,16,17]. 

Therefore, the current study aimed to validate the accuracy and repeatability of the London Atlas in a select SA sample of KwaZulu-Natal. 

## 2. Materials and Methods

### 2.1. Research Design and Data Acquisition 

Through consecutive sampling, a total of 760 digital panoramic radiographs (*n* = 760), within the 5.00 and 23.99 years of age range, were retrospectively reviewed in this cross-sectional study. These radiographs were captured using the Carestream Dental: CS 8100 unit and obtained from private dental practices within the KwaZulu-Natal region. The selected sample included 380 SA Black individuals and 380 SA Indian individuals equally distributed according to sex and age cohorts (yearly intervals). For statistical purposes, ten digital panoramic radiographs per demographic group was required to ensure an equal sample distribution. Consequently, the SA White and SA Colored population groups were excluded from the analysis as an insufficient number of radiographs were obtained. In line with the demographic conformation of the KwaZulu-Natal province, the investigated population groups (i.e., SA Black and SA Indian population groups) were reflective of the majority provincial population [18]. Within South Africa, there are four main population groups according to the “modern systems of racial classification”, which this study utilized to classify ancestry [19,20,21]. Chronological age was calculated by subtracting the date the radiograph was taken from the date of birth. All radiographs were assigned a number so the investigator was blinded to the demographic data at the time of the assessment. 

### 2.2. Ethical Consideration

This study received ethical approval from the institutional Biomedical Research Ethics Committee (BE: 405/17).

### 2.3. Inclusion and Exclusion Criteria

Digital panoramic radiographs with no impaction or extraction of dentition; no embryological defects; and/or no trauma or osteological pathologies to the maxillofacial region were included in this study. This study only included high-resolution radiographs that were free of any distortion.

## 3. Methodology

### 3.1. London Atlas of Human Tooth Development

Each radiograph was assessed utilizing the CS Imaging Software (Version: 7.0.20) and assigned a dental age in accordance with the London Atlas charts by AlQahtani et al. [7]. If the mandibular and maxillary dentition denoted inter-stage development, then an average age of the two stages was assigned. 

### 3.2. Intra-Observer and Inter-Observer Agreement

To ensure standardization of the methodology, two examiners jointly assessed ten radiographs. The principal investigator conducted the intra-observer reliability test on two separate occasions in four-week intervals to ensure accuracy and reproducibility. A second examiner re-analyzed forty-two digital panoramic radiographs using an identical methodology to certify inter-observer reliability and validity. The data were then analyzed using the intraclass correlation coefficient test.

### 3.3. Development of the KZN Atlas of Dental Development

**Figure 1:**
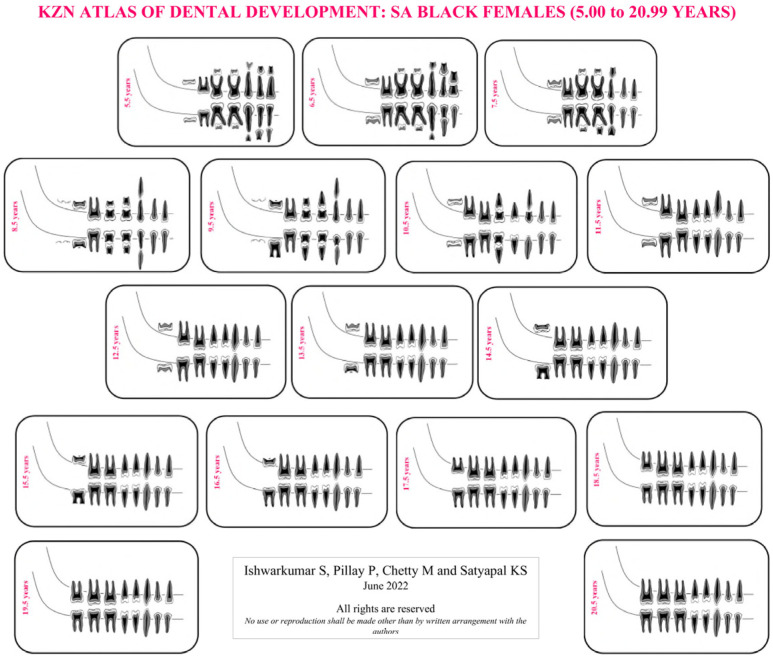
KZN Atlas of dental development for South African Black female.

**Figure 2:**
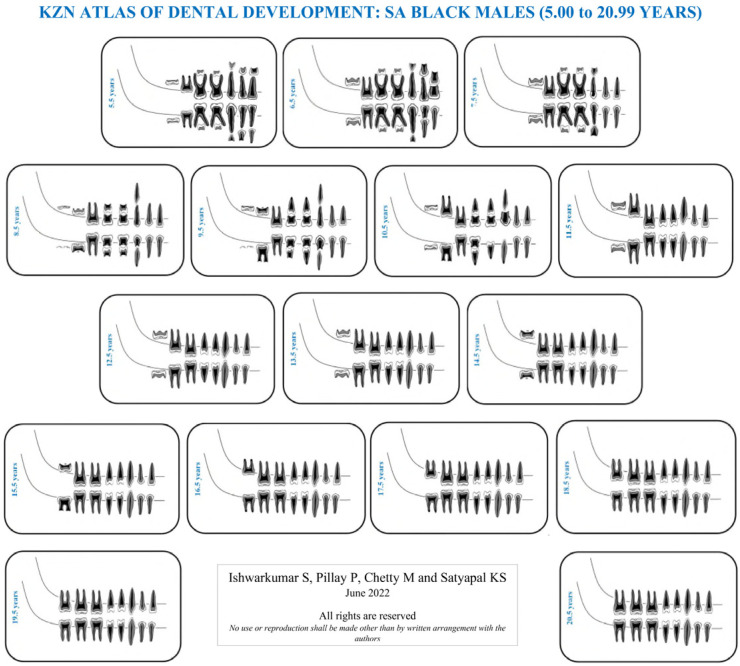
KZN Atlas of dental development for South African Black males.

**Figure 3:**
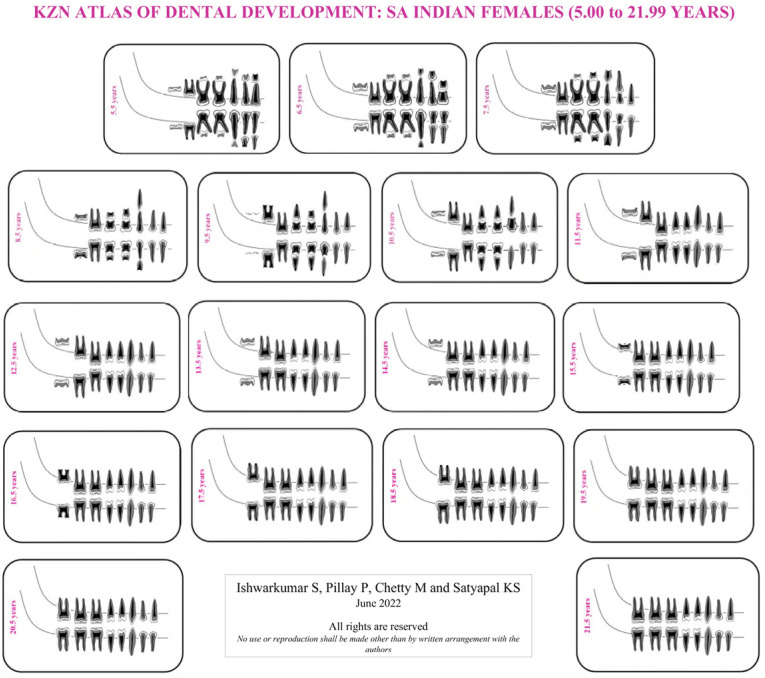
KZN Atlas of dental development for South African Indian females.

**Figure 4:**
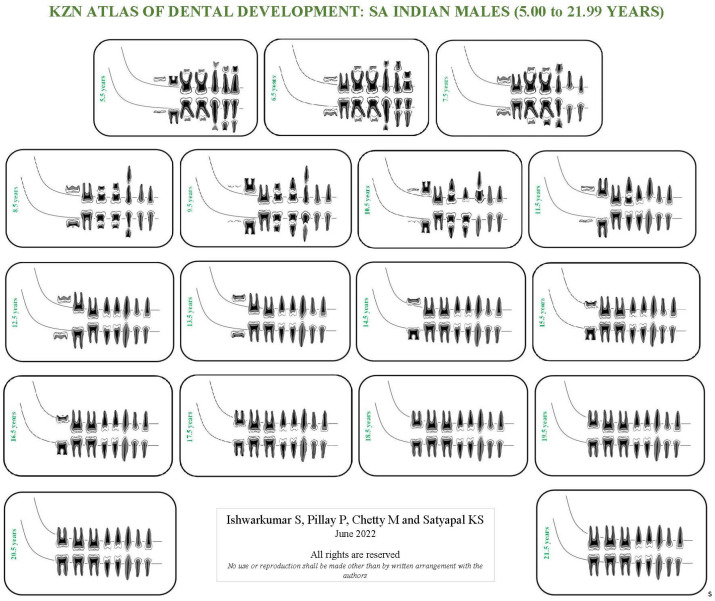
KZN Atlas of dental development for South African Indian males.

As the London Atlas was found to be not applicable to the selected KwaZulu-Natal population, population-specific reference charts and atlases were developed in this study. Four KZN Atlases were developed for SA Black and Indian male and female population groups of KwaZulu-Natal in accordance with the guidelines outlined by AlQahtani et al. [7]. This study evaluated the developmental stage of each tooth, in the right quadrant of the mandible and maxilla, on each radiograph in accordance with the Moorrees et al. [9] methodology. Thereafter, descriptive statistics in the form of frequency tables were categorically established for each age cohort, sex and population group sampled in this study. The dental developmental stage with the highest frequency (mode) in the aforementioned categories was assigned and utilized to develop the KZN Atlases (Appendix A: Table A1, Table A2, Table A3, Table A4, Table A5, Table A6, Table A7 and Table A8). If a category displayed an equal frequency for two or more developmental stages, then the more advanced stage was assigned [13]. The mode value was used instead of the median value for each stage, as not every variable has a median stage if there is an even number of stages [22]. Moreover, in accordance with AlQahtani et al. [7] and Esan and Schepartz [13], the age cohorts in the KZN Atlases were represented in mid-year intervals. The first author illustrated the individual teeth and assembled the KZN Atlases (Figure 1, Figure 2, Figure 3 and Figure 4).

### 3.4. Statistical Analysis

The statistical analysis was conducted utilizing the R Statistical Computing Software of the R Core Team 2020 (R version 3.6.3, Vienna, Austria). Descriptive statistics (frequencies, mean and standard deviation) were conducted. The performance of the London Atlas was assessed using the mean error (chronological age–dental age). In addition, a statistical comparison between the chronological age and the estimated dental age was conducted using the Wilcoxon test and paired samples t-test. A *p*-value less than 0.05 was considered statistically significant.

## 4. Results

### 4.1. Accuracy of the London Atlas of Human Tooth Development 

The London Atlas overestimated age with a mean difference of −0.85 to −1.26 years in the select SA sample of KwaZulu-Natal (Table 1). Notably, the London Atlas overestimated age more in males than females for both population groups (Table 1). Furthermore, the London Atlas overestimated age more in the selected SA Black (>1 year) sample than the SA Indian population group (<1 year) (Table 1). Moreover, a statistically significant difference between the chronological age and estimated dental age was found using the London Atlas for the selected sample (Table 1). Furthermore, the mean absolute error using the London Atlas for the total selected sample was 1.05 years. Therefore, this study developed population-specific charts for the SA Black and Indian population groups of KwaZulu-Natal to evaluate whether or not these charts would enhance the accuracy of age estimation within this region.

### 4.2. KZN Atlas of Dental Development

In the KwaZulu-Natal population, the most prevalent developmental stages recorded in the maxilla and mandible at 5.00 to 5.99 years were “R14” and “R34”, respectively (Appendix A: Table A1). Moreover, in the KZN Atlases, dental maturity occurred at 20.5 years in the SA Black population group and 21.5 years in the SA Indian population (Appendix A: Table A1, Table A2, Table A3, Table A4, Table A5, Table A6, Table A7 and Table A8). Furthermore, it was noted that the SA Black population group reached complete dental maturity a year before the selected SA Indian population group (Appendix A: Table A1, Table A2, Table A3, Table A4, Table A5, Table A6, Table A7 and Table A8).

### 4.3. Efficiency of the KZN Atlas of Dental Development

The efficiency of the KZN Atlases was tested on 60 digital panoramic radiographs that met the inclusion criteria. The mean chronological age of the sample was 15.48 ± 5.41 years, and the estimated dental age using the KZN Atlases and London Atlas was 15.30 ± 5.13 years and 16.84 ± 5.82 years, respectively. A mean difference of 0.18 years and mean absolute estimate of 0.57 years were recorded between the chronological age and estimated dental age for the KZN Atlases, which indicated a slight underestimation. However, this study’s statistically significant difference between the aforementioned parameters using the KZN Atlases was negligible (*p*-value = 0.1). Furthermore, a strong positive correlation between the chronological and dental ages was observed (R^2^ = 0.9862). 

### 4.4. Reliability and Validity

The intra- and inter-rater agreement in this study were 0.93 and 0.90, respectively, which denotes excellent and good agreement among the examiners according to guidelines outlined by Koo and Li [23]), respectively.

## 5. Discussion

Dental age estimation is considered to be one of the most reliable and accurate biological markers for age estimation, as dental development (calcification) is less affected by environmental conditions, diet and hormonal changes than skeletal bones [1,3,6]. In addition, teeth can be used for age estimation several years after death, as they are relatively indestructible [1]. In 2014, AlQahtani et al. [8] reported that the London Atlas more accurately assigned dental age than the Schour and Massler [24,25] and Ubelaker [26] atlases. This was due to the large sample size on which the atlas was based [27]. Sousa et al. [2] also mentioned that “more studies on different populations using the London Atlas would be fundamental to test how it performs in different population groups”. Most currently used dental age estimation methods have been developed on American and/or European population groups [7,24,26]. There are also limited studies on the applicability of these charts in a SA sample, specifically in the coastal regions of KwaZulu-Natal. KwaZulu-Natal is frequently affected by natural disasters (i.e., flooding and landslides), with more than 400 lives lost in the recent flooding in April 2022 [28]. Therefore, dental age methods are especially valuable to identify unknown remains. 

The London Atlas overestimated age in the selected KwaZulu-Natal population of SA. The mean chronological age was 14.50 ± 5.48 years for the overall sample, while the estimated dental age utilizing the London Atlas was 15.55 ± 5.86 years, with a mean difference of 1.05 years. This correlated with the findings of previous studies [1,3,4,6,26]. However, Alsudairi and AlQahtani [29] and Sankoung et al. [30] reported that the London Atlas underestimated age in the Saudi and Senegalese population, respectively. Moreover, Alshihri et al. [12] assessed the applicability of the London Atlas in the Saudi Arabian population; the authors reported that 65.5% of the dental age estimates were within 12 months of the subject’s chronological ages. In addition, Alshihri et al. [12] concluded that these differences emphasized the need to develop sex-specific charts. This study noted a statistically significant difference between the chronological age and estimated dental age, which correlated with Sharma and Wadhwan [3]. 

Notably, the overestimation in this study was less in females than males. This corroborated the findings of Pavlovic et al. [27]. This may be attributed to females reaching dental maturity earlier than males [2,16]. On the contrary, Sousa et al. [2] and Alkandiri et al. [6] noted a lower overestimation in males than females in Brazilian and Kuwaiti samples, respectively. 

Previous literary reports noted no statistically significant difference between the chronological age and estimated dental age on the right and left side of the jaw [22,27,31]. Pavlovic et al. [27] further elaborated that the side used for age estimation will not influence the results. Therefore, this study only evaluated the right side of the mandible and maxilla, which was in keeping with the studies conducted by AlQahtani et al. [7], Esan and Schepartz [13] and Putri et al. [22].

As a rule of thumb, estimated dental age is considered accurate in forensic and clinical cases if it is within six to twelve months of the chronological age; however, the former is preferred [13,32]. In the current study, the overestimation of dental age using the London Atlas in the selected sample was greater than six months. Sousa et al. [2] explained how the utilization of population-specific formulae may be essential for more accurate age estimation, thus highlighting the importance of establishing population-specific databases. According to McCloe et al. [33], the applicability of the London Atlas should be assessed in different ethnic groups to determine if inter-population variability exists. In addition, Esan and Schepartz [13] reported that dental development charts and tables based on mixed population samples may not be accurate for specific age estimation. In an attempt to mitigate the overestimation using the London Atlas, this study developed separate population- and sex-specific atlas charts and tables for the SA Black and Indian population groups of KwaZulu-Natal, with the latter recommended by Pavlovic et al. [27]. 

The median stages in the London Atlas for the central incisor at 5.5 years were “Ri” and “R14” in the maxilla and mandible, respectively [7]. In contrast, at this age range, development was more advanced in the KwaZulu-Natal population with stage “R14” in the maxilla and stage “R34” in the mandible. Moreover, the median age for dental maturity was 23.5 years in the London Atlas [7]. However, dental maturity occurred at 20.5 and 21.5 years in the SA Black and Indian populations in the KZN Atlases, respectively. In addition, the Indonesian Atlas of Tooth Development recorded dental maturity between 22 and 24 years in the Indonesian population [22]. Literary findings reported that children of African ancestry have advanced tooth development and emergence compared to those of European ancestry, hence attributing to the slightly earlier dental maturity in the SA Black population [13,34,35,36]. No inference can be made for the SA Indian population as the literature did not provide a comparison between African, European and Asian using the London Atlas.

The present study noted some differences between the WITS and KZN Atlases. The WITS Atlas reported dental maturity at 17.5 years [13], while in the KwaZulu-Natal population this occurred at 20.5 and 21.5 years in the SA Black and Indian populations, respectively. These differences may be attributed to variation among local sub-populations or regional differences [14,15,16]. The WITS Atlas was developed from a community-based study in the Gauteng province of South Africa, while this study was based on a KwaZulu-Natal population. This correlated with literary reports that noted differences in the development of dentition among individuals and different populations within the same geographical areas or cities [37,38]. Therefore, it is essential to adapt classification schemes to suit local sub-populations [38,39]. Moreover, Putri et al. [22] noted that even in the sample population group, variations in dental development may exist. These differences may be attributed to dietary and environmental factors, socioeconomic status and sociocultural differences [10].

The efficiencies of the KZN Atlases were tested on 60 additional digital panoramic radiographs. The mean chronological age of this sample was 15.48 ± 5.41 years, while the mean estimated dental age was 15.30 ± 5.13 years with a mean absolute estimate of 0.57 years. A slight statistically insignificant underestimation of 0.18 years was noted between the chronological age and estimated dental age, which denotes no statistical difference between the chronological age and estimated dental using the KZN Atlases (*p*-value = 0.1). The KZN Atlases are based on SA Black and Indian individuals aged between 5.00 and 21.99 years, with equal sample distribution between the different age cohorts, sex and population groups. The large sample distribution is one of the advantages to the KZN Atlases, in addition to being population-specific and sex-specific. The current study utilized 10 radiographs per age cohort for each sex and population group, which was similar to the London Atlas, which utilized 12 radiographs in each age cohort for males and females, respectively [7]. Alsudairi and AlQahtani [29] noted that, due to the large sample size used to establish the London Atlas, studies conducted on small sample sizes and uneven sample distribution may make it difficult to establish comparisons, thus justifying the comparisons drawn in this study.

This study recommends that the newly established KZN Atlases should be validated in different regions of South Africa and neighboring countries to assess their reproducibility. Furthermore, it is recommended that future studies investigate artificial intelligence for forensic age estimation, viz., machine learning and/or neural networks, using this methodology. The investigators acknowledge that a limitation of this study was excluding the SA Colored and SA White populations from the analysis. However, insufficient data were obtained to ensure statistically reliability for the aforementioned groups. This is attributed to the demographic composition of KwaZulu-Natal with the SA Black and SA Indian population groups being predominately located within this region [18]. In addition, this study was unable to access radiographs of children younger than 5 years old, since in South Africa it is not recommended to expose young children to radiation useless it is imperative for specific medical procedures. 

## 6. Conclusions

The London Atlas significantly overestimated age by −0.85 to −1.26 years in the selected SA Black and Indian population groups of KwaZulu-Natal (*p*-value > 0.05). Therefore, this study created the KZN Atlases, which are population- and sex-specific charts for the aforementioned populations groups of KwaZulu-Natal. The KZN Atlases were found to be more accurate than the London Atlas in the selected sample, as the estimated dental age derived from the KZN Atlases were closest to the chronological age. Moreover, insignificant statistical differences between the chronological age and estimated dental ages using the KZN Atlases were found in this study. Therefore, the KZN Atlases could be utilized in forensic, medico-legal and civil cases in KwaZulu-Natal to enhance the accuracy of dental age estimation. 

## Figures and Tables

**Table 1:** Dental age estimation using the London Atlas in the select KwaZulu-Natal sample (in years).

Population Group	Sex	Sample Size (*n*)	Chronological Age (CA) ± SD	Dental Age (DA) ± SD	* Mean Error (CA–DA)	Mean Absolute Error	*p*-Value
SA Black	Female	190	14.50 ± 5.47	15.63 ± 5.82	−1.13	1.28	<0.001
SA Black	Male	190	14.49 ± 5.48	15.75 ± 5.89	−1.26	1.40	<0.001
SA Indian	Female	190	14.51 ± 5.44	15.36 ± 5.64	−0.85	1.10	<0.001
SA Indian	Male	190	14.48 ± 5.47	15.46 ± 6.02	−0.98	1.21	<0.001
Total Sample		760	14.50 ± 5.48	15.55 ± 5.86	−1.05	1.25	<0.001

* Mean error (CA-DA): A negative value indicated overestimations and a positive value indicates underestimation.

## Appendix A

**Table A1:** Frequencies of the tooth development stages of the central incisors for the SA Black and Indian population groups.

Maxillary Central Incisors (I1)								Mandibular Central Incisors (I1)							
Age Range	Female			Male			Grand Total	Age Range	Female			Male			Grand Total
Tooth Stages	Black	Indian	Total	Black	Indian	Total		Tooth Stages	Black	Indian	Total	Black	Indian	Total	
**5.00–5.99**	**10**	**10**	**20**	**10**	**10**	**20**	**40**	**5.00–5.99**	**10**	**10**	**20**	**10**	**10**	**20**	**40**
Cr3/4					1	1	1	Ri					1	1	1
Crc	2	3	5	2	3	5	10	R1/4		1	1	2	2	4	5
Ri	1	2	3	4	1	5	8	R1/2	2	3	5	1	3	4	9
R1/4	3	3	6	1	3	4	10	R3/4	8	5	13	6	4	10	23
R1/2	4		4	1	2	3	7	Rc		1	1	1		1	2
R3/4		2	2	2		2	4								
**6.00–6.99**	**10**	**10**	**20**	**10**	**10**	**20**	**40**	**6.00–6.99**	**10**	**10**	**20**	**10**	**10**	**20**	**40**
Crc					2	2	2	R1/4					1	1	1
R1/4	1	4	5	2	4	6	11	R1/2	1	1	2	3	1	4	6
R1/2	5	2	7	5	1	6	13	R3/4	2	5	7	2	8	10	17
R3/4	1	3	4	3	3	6	10	Rc	5	4	9	5		5	14
Rc	3	1	4				4	Ac	2		2				2
**7.00–7.99**	**10**	**10**	**20**	**10**	**10**	**20**	**40**	**7.00–7.99**	**10**	**10**	**20**	**10**	**10**	**20**	**40**
Crc					1	1	1	R3/4		1	1	1	1	2	3
R1/2				1	1	2	2	Rc	3	7	10	3	5	8	18
R3/4	5	6	11	5	1	6	17	A1/2	5	2	7	5	3	8	15
Rc	4	4	8	2	7	9	17	Ac	2		2	1	1	2	4
A1/2	1		1	2		2	3								
**8.00–8.99**	**10**	**10**	**20**	**10**	**10**	**20**	**40**	**8.00–8.99**	**10**	**10**	**20**	**10**	**10**	**20**	**40**
R3/4	2		2		3	3	5	R3/4					1	1	1
Rc	4	8	12	3	7	10	22	Rc	3	2	5	2		2	7
A1/2	2	2	4	5		5	9	A1/2	5	8	13	3	5	8	21
Ac	2		2	2		2	4	Ac	2		2	5	4	9	11
**9.00–9.99**	**10**	**10**	**20**	**10**	**10**	**20**	**40**	**9.00–9.99**	**10**	**10**	**20**	**10**	**10**	**20**	**40**
R3/4				1		1	1	R3/4				1		1	1
Rc	2	4	6	1	7	8	14	Rc				1	1	2	2
A1/2	5	1	6	2	2	4	10	A1/2	2	2	4		2	2	6
Ac	3	5	8	6	1	7	15	Ac	8	8	16	8	7	15	31
**10.00–10.99**	**10**	**10**	**20**	**10**	**10**	**20**	**40**	**10.00–10.99**	**10**	**10**	**20**	**10**	**10**	**20**	**40**
Rc				2		2	2	Rc				1		1	1
A1/2	3	2	5	1	2	3	8	A1/2	1	1	2	1		1	3
Ac	7	8	15	7	8	15	30	Ac	9	9	18	8	10	18	36
**11.00–11.99**	**10**	**10**	**20**	**10**	**10**	**20**	**40**	**11.00–11.99**	**10**	**10**	**20**	**10**	**10**	**20**	**40**
A1/2		1	1				1	Ac	10	10	20	10	10	20	40
Ac	10	9	19	10	10	20	39								
**12.00–12.99 to** **23.00–23.99**	**120**	**120**	**240**	**120**	**120**	**240**	**480**	**12.00–12.99 to** **23.00–23.99**	**120**	**120**	**240**	**120**	**120**	**240**	**480**
Ac	120	120	240	120	120	240	480	Ac	120	120	240	120	120	240	480

Key: Ci—initial cusp formation; Cco—coalescence of cusp; Coc—cusp outline complete; Cr1/2—crown half completed; Cr3/4—crown three-quarters completed; Crc—crown completed; Ri—initial root formation; R1/4—root length less than crown length; R1/2—root length equals crown length; R3/4—three-quarters of root length developed; Rc—root length completed; A1/2—apex closed with wide periodontal ligament width; Ac—apex closed with normal periodontal ligament width.

**Table A2:** Frequencies of the tooth development stages of the lateral incisors for the SA Black and Indian population groups.

Maxillary Lateral Incisors (I2)								Mandibular Lateral Incisors (I2)							
Age Range	Female			Male			Grand Total	Male	Female			Male			Grand Total
Tooth Stages	Black	Indian	Total	Black	Indian	Total		Tooth Stages	Black	Indian	Total	Black	Indian	Total	
**5.00–5.99**	**10**	**10**	**20**	**10**	**10**	**20**	**40**	**5.00–5.99**	**10**	**10**	**20**	**10**	**10**	**20**	**40**
Cr1/2					1	1	1	Ri		1	1	1	3	4	5
Cr3/4				1	1	2	2	R1/4	2	1	3	2	1	3	6
Crc	3	5	8	4	6	10	18	R1/2	5	5	10	5	4	9	19
Ri	1	2	3		1	1	4	R3/4	3	3	6	2	2	4	10
R1/4	4	1	5	3	1	4	9								
R1/2	2	1	3	1		1	4								
R3/4		1	1	1		1	2								
**6.00–6.99**	**10**	**10**	**20**	**10**	**10**	**20**	**40**	**6.00–6.99**	**10**	**10**	**20**	**10**	**10**	**20**	**40**
Crc				2	3	5	5	R1/4		1	1		1	1	2
Ri	1		1	1	1	2	3	R1/2	1	2	3	3	4	7	10
R1/4	3	5	8	3	1	4	12	R3/4	5	5	10	4	5	9	19
R1/2	5	2	7	4	4	8	15	Rc	4	2	6	3		3	9
R3/4	1	3	4		1	1	5								
**7.00–7.99**	**10**	**10**	**20**	**10**	**10**	**20**	**40**	**7.00–7.99**	**10**	**10**	**20**	**10**	**10**	**20**	**40**
Crc					2	2	2	R1/2		1	1	1		1	2
R1/4					1	1	1	R3/4		4	4	2	2	4	8
R1/2	2	4	6	2	1	3	9	Rc	8	5	13	5	8	13	26
R3/4	2	4	6	5	4	9	15	A1/2	1		1	2		2	3
Rc	6	2	8	3	2	5	13	Ac	1		1				1
**8.00–8.99**	**10**	**10**	**20**	**10**	**10**	**20**	**40**	**8.00–8.99**	**10**	**10**	**20**	**10**	**10**	**20**	**40**
Crc					1	1	1	R3/4		1	1	1	1	2	3
R1/2	1	1	2		1	1	3	Rc	3	5	8	3	4	7	15
R3/4	2		2	3	2	5	7	A1/2	6	4	10	4	3	7	17
Rc	5	8	13	2	6	8	21	Ac	1		1	2	2	4	5
A1/2	1	1	2	5		5	7								
Ac	1		1				1								
**9.00–9.99**	**10**	**10**	**20**	**10**	**10**	**20**	**40**	**9.00–9.99**	**10**	**10**	**20**	**10**	**10**	**20**	**40**
R1/2					1	1	1	Rc	1	1	2	3	2	5	7
R3/4				1	2	3	3	A1/2	6	5	11	2	5	7	18
Rc	3	5	8	3	5	8	16	Ac	3	4	7	5	3	8	15
A1/2	5	1	6	2	1	3	9								
Ac	2	4	6	4	1	5	11								
**10.00–10.99**	**10**	**10**	**20**	**10**	**10**	**20**	**40**	**10.00–10.99**	**10**	**10**	**20**	**10**	**10**	**20**	**40**
Rc	1		1	2	2	4	5	R3/4				1		1	1
A1/2	2	4	6	3	3	6	12	A1/2	1	2	3	2		2	5
Ac	7	6	13	5	5	10	23	Ac	9	8	17	7	10	17	34
**11.00–11.99**	**10**	**10**	**20**	**10**	**10**	**20**	**40**	**11.00–11.99**	**10**	**10**	**20**	**10**	**10**	**20**	**40**
Rc				1		1	1	A1/2		1	1				1
A1/2		2	2		1	1	3	Ac	10	9	19	10	10	20	39
Ac	10	8	18	9	9	18	36								
**12.00–12.99 to** **23.00–23.99**	**120**	**120**	**240**	**120**	**120**	**240**	**480**	**12.00–12.99 to** **23.00–23.99**	**120**	**120**	**240**	**120**	**120**	**240**	**480**
Ac	120	120	240	120	120	240	480	Ac	120	120	240	120	120	240	480

Key: Ci—initial cusp formation; Cco—coalescence of cusp; Coc—cusp outline complete; Cr1/2—crown half completed; Cr3/4—crown three-quarters completed; Crc—crown completed; Ri—initial root formation; R1/4—root length less than crown length; R1/2—root length equals crown length; R3/4—three-quarters of root length developed; Rc—root length completed; A1/2—apex closed with wide periodontal ligament width; Ac—apex closed with normal periodontal ligament width.

**Table A3:** Frequencies of the tooth development stages of the canines for the SA Black and Indian population groups.

Maxillary Canines (C)								Mandibular Canines (C)							
Age Range	Female			Male			Grand Total	Age Range	Female			Male			Grand Total
Tooth Stages	Black	Indian	Total	Black	Indian	Total		Tooth Stages	Black	Indian	Total	Black	Indian	Total	
**5.00–5.99**	**10**	**10**	**20**	**10**	**10**	**20**	**40**	**5.00–5.99**	**10**	**10**	**20**	**10**	**10**	**20**	**40**
Cr1/2				1		1	1	Cr1/2					1	1	1
Cr3/4					1	1	1	Cr3/4				1		1	1
Crc	8	8	16	6	9	15	31	Crc	2	5	7	2	7	9	16
Ri				3		3	3	Ri	3	2	5	5	2	7	12
R1/4	1	1	2				2	R1/4	4	2	6	2		2	8
R1/2	1	1	2				2	R1/2	1		1				1
								R3/4		1	1				1
**6.00–6.99**	**10**	**10**	**20**	**10**	**10**	**20**	**40**	**6.00–6.99**	**10**	**10**	**20**	**10**	**10**	**20**	**40**
Crc	1	4	5	3	4	7	12	Crc	1		1	3	4	7	8
Ri		1	1	4	2	6	7	Ri	2		2	2	1	3	5
R1/4	8	4	12	2	4	6	18	R1/4	7	6	13	4	4	8	21
R1/2	1		1				1	R1/2		3	3	1		1	4
R3/4		1	1	1		1	2	R3/4		1	1		1	1	2
**7.00–7.99**	**10**	**10**	**20**	**10**	**10**	**20**	**40**	**7.00–7.99**	**10**	**10**	**20**	**10**	**10**	**20**	**40**
Crc					3	3	3	Cr3/4				1		1	1
Ri	2		2				2	Crc					1	1	1
R1/4	3	2	5	5	3	8	13	Ri					2	2	2
R1/2	3	5	8	3	3	6	14	R1/4	3	1	4	3	4	7	11
R3/4	2	3	5	1	1	2	7	R1/2	4	5	9	3	1	4	13
Rc				1		1	1	R3/4	3	4	7	2	2	4	11
								Rc				1		1	1
**8.00–8.99**	**10**	**10**	**20**	**10**	**10**	**20**	**40**	**8.00–8.99**	**10**	**10**	**20**	**10**	**10**	**20**	**40**
Crc					3	3	3	Crc				1		1	1
Ri				1		1	1	R1/4	1	3	4	1	2	3	7
R1/4		2	2	2	2	4	6	R1/2	2	4	6	3	5	8	14
R1/2	1	4	5	2	1	3	8	R3/4	7	3	10	5	3	8	18
R3/4	9	4	13	5	4	9	22								
**9.00–9.99**	**10**	**10**	**20**	**10**	**10**	**20**	**40**	**9.00–9.99**	**10**	**10**	**20**	**10**	**10**	**20**	**40**
R1/4		1	1				1	Ri					1	1	1
R1/2	4	1	5	1	3	4	9	R1/4		1	1				1
R3/4	6	7	13	9	7	16	29	R1/2	2	1	3	1		1	4
Rc		1	1				1	R3/4	5	5	10	9	7	16	26
								Rc	3	3	6		2	2	8
**10.00–10.99**	**10**	**10**	**20**	**10**	**10**	**20**	**40**	**10.00–10.99**	**10**	**10**	**20**	**10**	**10**	**20**	**40**
R1/2	1		1	1		1	2	R1/2	1		1	1		1	2
R3/4	5	6	11	6	7	13	24	R3/4	4	6	10	6	8	14	24
Rc	4	4	8	3	3	6	14	Rc	5	4	9	3	2	5	14
**11.00–11.99**	**10**	**10**	**20**	**10**	**10**	**20**	**40**	**11.00–11.99**	**10**	**10**	**20**	**10**	**10**	**20**	**40**
R3/4	2	2	4	1	5	6	10	R3/4	1	2	3	2	4	6	9
Rc	7	7	14	9	5	14	28	Rc	8	7	15	8	6	14	29
A1/2	1	1	2				2	A1/2	1	1	2				2
**12.00–12.99**	**10**	**10**	**20**	**10**	**10**	**20**	**40**	**12.00–12.99**	**10**	**10**	**20**	**10**	**10**	**20**	**40**
R3/4	1		1	1	3	4	5	R3/4	1	1	2		2	2	4
Rc	7	9	16	9	6	15	31	Rc	6	8	14	10	7	17	31
A1/2	2		2		1	1	3	A1/2	2	1	3		1	1	4
Ac		1	1				1	Ac	1		1				1
**13.00–13.99**	**10**	**10**	**20**	**10**	**10**	**20**	**40**	**13.00–13.99**	**10**	**10**	**20**	**10**	**10**	**20**	**40**
R3/4					1	1	1	R3/4	2		2		1	1	3
Rc	9	9	18	5	7	12	30	Rc	8	8	16	4	8	12	28
A1/2		1	1	3	2	5	6	A1/2		2	2	5	1	6	8
Ac	1		1	2		2	3	Ac				1		1	1
**14.00–14.99**	**10**	**10**	**20**	**10**	**10**	**20**	**40**	**14.00–14.99**	**10**	**10**	**20**	**10**	**10**	**20**	**40**
Rc	4	3	7	3	3	6	13	Rc	4	3	7	3	3	6	13
A1/2	5	5	10	5	4	9	19	A1/2	4	4	8	1	4	5	13
Ac	1	2	3	2	3	5	8	Ac	2	3	5	6	3	9	14
**15.00–15.99**	**10**	**10**	**20**	**10**	**10**	**20**	**40**	**15.00–15.99**	**10**	**10**	**20**	**10**	**10**	**20**	**40**
Rc		2	2		1	1	3	Rc		1	1		1	1	2
A1/2	3	2	5	1	3	4	9	A1/2	3	4	7	1	3	4	11
Ac	7	6	13	9	6	15	28	Ac	7	5	12	9	6	15	27
**16.00–16.99 to 23.00–23.99**	**80**	**80**	**160**	**80**	**80**	**160**	**320**	**16.00–16.99 to 23.00–23.99**	**80**	**80**	**160**	**80**	**80**	**160**	**320**
Ac	80	80	160	80	80	160	320	Ac	80	80	160	80	80	160	320

Key: Ci—initial cusp formation; Cco—coalescence of cusp; Coc—cusp outline complete; Cr1/2—crown half completed; Cr3/4—crown three-quarters completed; Crc—crown completed; Ri—initial root formation; R1/4—root length less than crown length; R1/2—root length equals crown length; R3/4—three-quarters of root length developed; Rc—root length completed; A1/2—apex closed with wide periodontal ligament width; Ac—apex closed with normal periodontal ligament width.

**Table A4:** Frequencies of the tooth development stages of the first premolar for the SA Black and Indian population groups.

Maxillary First Premolar (PM1)								Mandibular First Premolar (PM1)							
Age Range	Female			Male			Grand Total	Age range	Female			Male			Grand Total
Tooth Stages	Black	Indian	Total	Black	Indian	Total		Tooth stages	Black	Indian	Total	Black	Indian	Total	
**5.00–5.99**	**10**	**10**	**20**	**10**	**10**	**20**	**40**	**5.00–5.99**	**10**	**10**	**20**	**10**	**10**	**20**	**40**
Cr1/2					3	3	3	Cr1/2					2	2	2
Cr3/4		2	2	1		1	3	Cr3/4		1	1	1		1	2
Crc	10	8	18	9	7	16	34	Crc	10	9	19	8	8	16	35
								Ri				1		1	1
**6.00–6.99**	**10**	**10**	**20**	**10**	**10**	**20**	**40**	**6.00–6.99**	**10**	**10**	**20**	**10**	**10**	**20**	**40**
Cr3/4	1		1		1	1	2	Cr3/4					1	1	1
Crc	8	8	16	9	8	17	33	Crc	5	7	12	7	6	13	25
Ri	1		1				1	Ri	5	1	6	2	2	4	10
R1/4		2	2	1	1	2	4	R1/4		2	2	1	1	2	4
**7.00–7.99**	**10**	**10**	**20**	**10**	**10**	**20**	**40**	**7.00–7.99**	**10**	**10**	**20**	**10**	**10**	**20**	**40**
Cr1/2					1	1	1	Crc	2	2	4	2	3	5	9
Crc	2	3	5	6	5	11	16	Ri	2	3	5	3	2	5	10
Ri	4		4				4	R1/4	2	3	5	2	4	6	11
R1/4		4	4	2	2	4	8	R1/2	3	2	5	1	1	2	7
R1/2	3	3	6		2	2	8	R3/4				1		1	1
Rc	1		1	2		2	3	Rc	1		1	1		1	2
**8.00–8.99**	**10**	**10**	**20**	**10**	**10**	**20**	**40**	**8.00–8.99**	**10**	**10**	**20**	**10**	**10**	**20**	**40**
Crc		1	1	1	1	2	3	Crc				1		1	1
Ri					1	1	1	Ri					1	1	1
R1/4		4	4	4	2	6	10	R1/4	1	7	8	4	5	9	17
R1/2	6	4	10	2	5	7	17	R1/2	6	2	8	3	1	4	12
R3/4	4	1	5	3	1	4	9	R3/4	3	1	4	2	3	5	9
**9.00–9.99**	**10**	**10**	**20**	**10**	**10**	**20**	**40**	**9.00–9.99**	**10**	**10**	**20**	**10**	**10**	**20**	**40**
								Ri		1	1				1
R1/4				1		1	1	R1/4	1	1	2				2
R1/2	4	5	9	3	2	5	14	R1/2	4	2	6	3	5	8	14
R3/4	5	2	7	5	8	13	20	R3/4	4	4	8	6	5	11	19
Rc	1	3	4	1		1	5	Rc	1	2	3	1		1	4
**10.00–10.99**	**10**	**10**	**20**	**10**	**10**	**20**	**40**	**10.00–10.99**	**10**	**10**	**20**	**10**	**10**	**20**	**40**
R1/2				1	2	3	3	R1/2				1	2	3	3
R3/4	3	1	4	4	4	8	12	R3/4	3	3	6	5	4	9	15
Rc	7	9	16	4	4	8	24	Rc	7	7	14	4	4	8	22
Ac				1		1	1								
**11.00–11.99**	**10**	**10**	**20**	**10**	**10**	**20**	**40**	**11.00–11.99**	**10**	**10**	**20**	**10**	**10**	**20**	**40**
R3/4					1	1	1	R3/4		1	1		1	1	2
Rc	7	7	14	7	6	13	27	Rc	4	8	12	8	7	15	27
A1/2	3	2	5	2	2	4	9	A1/2	5	1	6	2	2	4	10
Ac		1	1	1	1	2	3	Ac	1		1				1
**12.00–12.99**	**10**	**10**	**20**	**10**	**10**	**20**	**40**	**12.00–12.99**	**10**	**10**	**20**	**10**	**10**	**20**	**40**
								R3/4		1	1				1
Rc	2	3	5	1	6	7	12	Rc	1		1	3		3	4
A1/2	6	5	11	5	1	6	17	A1/2	8	6	14	6	6	12	26
Ac	2	2	4	4	3	7	11	Ac	1	3	4	1	4	5	9
**13.00–13.99**	**10**	**10**	**20**	**10**	**10**	**20**	**40**	**13.00–13.99**	**10**	**10**	**20**	**10**	**10**	**20**	**40**
Rc				2		2	2	Rc		1	1	1		1	2
A1/2	4	3	7	2	1	3	10	A1/2	4	3	7	3	1	4	11
Ac	6	7	13	6	9	15	28	Ac	6	6	12	6	9	15	27
**14.00–14.99**	**10**	**10**	**20**	**10**	**10**	**20**	**40**	**14.00–14.99**	**10**	**10**	**20**	**10**	**10**	**20**	**40**
Rc				1		1	1	Rc				1		1	1
A1/2	1	1	2	1		1	3	A1/2		1	1				1
Ac	9	9	18	8	10	18	36	Ac	10	9	19	9	10	19	38
**15.00–15.99**	**10**	**10**	**20**	**10**	**10**	**20**	**40**	**15.00–15.99**	**10**	**10**	**20**	**10**	**10**	**20**	**40**
A1/2				1		1	1								
Ac	10	10	20	9	10	19	39	Ac	10	10	20	10	10	20	40
**16.00–16.99 to 23.00–23.99**	**80**	**80**	**160**	**80**	**80**	**160**	**320**	**16.00–16.99 to 23.00–23.99**	**80**	**80**	**160**	**80**	**80**	**160**	**320**
Ac	80	80	160	80	80	160	320	Ac	80	80	160	80	80	160	320

Key: Ci—initial cusp formation; Cco—Coalescence of cusp; Coc—Cusp outline complete; Cr1/2—crown half completed; Cr3/4—crown three-quarters completed; Crc—crown completed; Ri—initial root formation; R1/4—root length less than crown length; R1/2—root length equals crown length; R3/4—three-quarters of root length developed; Rc—root length completed; A1/2—apex closed with wide periodontal ligament width; Ac—apex closed with normal periodontal ligament width.

**Table A5:** Frequencies of the tooth development stages of the second premolar for the SA Black and Indian population groups.

Maxillary Second Premolar (PM2)								Mandibular Second Premolar (PM2)							
Age Range	Female			Male			Grand Total	Age Range	Female			Male			Grand Total
Tooth Stages	Black	Indian	Total	Black	Indian	Total		Tooth Stages	Black	Indian	Total	Black	Indian	Total	
**5.00–5.99**	**10**	**10**	**20**	**10**	**10**	**20**	**40**	**5.00–5.99**	**10**	**10**	**20**	**10**	**10**	**20**	**40**
Cr1/2		5	5		3	3	8	Cr1/2	1	3	4		3	3	7
Cr3/4	2	2	4	1	3	4	8	Cr3/4	1		1	1	3	4	5
Crc	8	3	11	9	4	13	24	Crc	8	7	15	9	4	13	28
**6.00–6.99**	**10**	**10**	**20**	**10**	**10**	**20**	**40**	**6.00–6.99**	**10**	**10**	**20**	**10**	**10**	**20**	**40**
Cr1/2	1	2	3	1	3	4	7	Cr1/2		1	1				1
Cr3/4				2	1	3	3	Cr3/4	2		2	1	1	2	4
Crc	9	7	16	6	6	12	28	Crc	7	8	15	8	7	15	30
Ri		1	1				1	Ri	1		1	1	1	2	3
R1/4				1		1	1	R1/4		1	1		1	1	2
**7.00–7.99**	**10**	**10**	**20**	**10**	**10**	**20**	**40**	**7.00–7.99**	**10**	**10**	**20**	**10**	**10**	**20**	**40**
Coc					1	1	1	Crc	3	2	5	5	4	9	14
Crc	6	6	12	7	5	12	24	Ri	4	2	6	2	3	5	11
Ri	1	1	2		2	2	4	R1/4		5	5	1	3	4	9
R1/4	1	3	4	1	2	3	7	R1/2	2	1	3				3
R1/2	1		1				1	R3/4				1		1	1
Rc	1		1	2		2	3	Rc	1		1	1		1	2
**8.00–8.99**	**10**	**10**	**20**	**10**	**10**	**20**	**40**	**8.00–8.99**	**10**	**10**	**20**	**10**	**10**	**20**	**40**
Crc		4	4	2	3	5	9	Crc		1	1	2	1	3	4
Ri	2		2	1	1	2	4	Ri					3	3	3
R1/4	4	5	9	3	3	6	15	R1/4	2	8	10	5	4	9	19
R1/2	2		2	2	2	4	6	R1/2	8		8	2	2	4	12
R3/4	2	1	3	2	1	3	6	R3/4		1	1	1		1	2
**9.00–9.99**	**10**	**10**	**20**	**10**	**10**	**20**	**40**	**9.00–9.99**	**10**	**10**	**20**	**10**	**10**	**20**	**40**
Crc		3	3	1	2	3	6	Ri		2	2	1		1	3
R1/4	2	1	3	1	1	2	5	R1/4	2	1	3	2	2	4	7
R1/2	6	1	7	3	4	7	14	R1/2	4	3	7	4	5	9	16
R3/4	1	3	4	4	3	7	11	R3/4	4	3	7	2	3	5	12
Rc	1	2	3	1		1	4	Rc		1	1	1		1	2
**10.00–10.99**	**10**	**10**	**20**	**10**	**10**	**20**	**40**	**10.00–10.99**	**10**	**10**	**20**	**10**	**10**	**20**	**40**
Ri					1	1	1	R1/4		1	1	1		1	2
R1/2	3	1	4	3	4	7	11	R1/2	2	1	3	1	3	4	7
R3/4	4	3	7	3	5	8	15	R3/4	4	5	9	7	7	14	23
Rc	3	6	9	4		4	13	Rc	4	3	7	1		1	8
**11.00–11.99**	**10**	**10**	**20**	**10**	**10**	**20**	**40**	**11.00–11.99**	**10**	**10**	**20**	**10**	**10**	**20**	**40**
R1/2		1	1				1	R1/2		2	2	1		1	3
R3/4	1	2	3	2	4	6	9	R3/4	2	3	5	2	3	5	10
Rc	8	5	13	6	5	11	24	Rc	7	5	12	6	6	12	24
A1/2	1	2	3	1	1	2	5	A1/2	1		1	1	1	2	3
Ac				1		1	1								
**12.00–12.99**	**10**	**10**	**20**	**10**	**10**	**20**	**40**	**12.00–12.99**	**10**	**10**	**20**	**10**	**10**	**20**	**40**
								R1/2					1	1	1
R3/4		1	1		3	3	4	R3/4		3	3	1	1	2	5
Rc	5	6	11	3	4	7	18	Rc	3	3	6	5	3	8	14
A1/2	4	2	6	4		4	10	A1/2	7	3	10	3	3	6	16
Ac	1	1	2	3	3	6	8	Ac		1	1	1	2	3	4
**13.00–13.99**	**10**	**10**	**20**	**10**	**10**	**20**	**40**	**13.00–13.99**	**10**	**10**	**20**	**10**	**10**	**20**	**40**
Rc		1	1	2		2	3	Rc	1	1	2	1		1	3
A1/2	6	2	8	5	1	6	14	A1/2	5	2	7	5	2	7	14
Ac	4	7	11	3	9	12	23	Ac	4	7	11	4	8	12	23
**14.00–14.99**	**10**	**10**	**20**	**10**	**10**	**20**	**40**	**14.00–14.99**	**10**	**10**	**20**	**10**	**10**	**20**	**40**
Rc	1	1	2	3		3	5	Rc		1	1		1	1	2
A1/2		1	1	2		2	3	A1/2		1	1	3		3	4
Ac	9	8	17	5	10	15	32	Ac	10	8	18	7	9	16	34
**15.00–15.99**	**10**	**10**	**20**	**10**	**10**	**20**	**40**	**15.00–15.99**	**10**	**10**	**20**	**10**	**10**	**20**	**40**
								Rc		1	1				1
A1/2				2		2	2	A1/2		1	1				1
Ac	10	10	20	8	10	18	38	Ac	10	8	18	10	10	20	38
**16.00–16.99 to 23.00–23.99**	**80**	**80**	**160**	**80**	**80**	**160**	**320**	**16.00–16.99 to 23.00–23.99**	**80**	**80**	**160**	**80**	**80**	**160**	**320**
Ac	80	80	160	80	80	160	320	Ac	80	80	160	80	80	160	320

Key: Ci—initial cusp formation; Cco—coalescence of cusp; Coc—cusp outline complete; Cr1/2—crown half completed; Cr3/4—crown three-quarters completed; Crc—crown completed; Ri—initial root formation; R1/4—root length less than crown length; R1/2—root length equals crown length; R3/4—three-quarters of root length developed; Rc—root length completed; A1/2—apex closed with wide periodontal ligament width; Ac—apex closed with normal periodontal ligament width.

**Table A6:** Frequencies of the tooth development stages of the first molar for the SA Black and Indian population groups.

Maxillary First Molar (M1)								Mandibular First Molar (M1)							
Age Range	Female			Male			Grand Total	Age Range	Female			Male			Grand Total
Tooth Stages	Black	Indian	Total	Black	Indian	Total		Tooth Stages	Black	Indian	Total	Black	Indian	Total	
**5.00–5.99**	**10**	**10**	**20**	**10**	**10**	**20**	**40**	**5.00–5.99**	**10**	**10**	**20**	**10**	**10**	**20**	**40**
Ri				1	3	4	4	Ri					1	1	1
R1/4	1	1	2	2		2	4	R1/4				1	2	3	3
R1/2	2	1	3	2	3	5	8	R1/2	3	1	4	3	2	5	9
R3/4	5	5	10	3	2	5	15	R3/4	4	5	9	5	4	9	18
Rc	2	3	5	2	2	4	9	Rc	3	4	7	1	1	2	9
**6.00–6.99**	**10**	**10**	**20**	**10**	**10**	**20**	**40**	**6.00–6.99**	**10**	**10**	**20**	**10**	**10**	**20**	**40**
R1/4					1	1	1	R1/2				1	1	2	2
R1/2				3		3	3	R3/4	4	1	5	4	2	6	11
R3/4	4	1	5	2	3	5	10	Rc	5	7	12	5	7	12	24
Rc	5	7	12	5	6	11	23	A1/2	1	2	3				3
A1/2	1	2	3				3								
**7.00–7.99**	**10**	**10**	**20**	**10**	**10**	**20**	**40**	**7.00–7.99**	**10**	**10**	**20**	**10**	**10**	**20**	**40**
R3/4		1	1				1	Rc	6	6	12	6	6	12	24
Rc	6	5	11	9	7	16	27	A1/2	3	4	7	3	4	7	14
A1/2	4	4	8	1	3	4	12	Ac	1		1	1		1	2
**8.00–8.99**	**10**	**10**	**20**	**10**	**10**	**20**	**40**	**8.00–8.99**	**10**	**10**	**20**	**10**	**10**	**20**	**40**
R3/4				1		1	1	Rc	3	1	4	4	2	6	10
Rc	4	1	5	4	3	7	12	A1/2	4	9	13	6	6	12	25
A1/2	5	9	14	5	6	11	25	Ac	3		3		2	2	5
Ac	1		1		1	1	2								
**9.00–9.99**	**10**	**10**	**20**	**10**	**10**	**20**	**40**	**9.00–9.99**	**10**	**10**	**20**	**10**	**10**	**20**	**40**
R3/4				1		1	1	Rc				2		2	2
Rc		1	1				1	A1/2	6	6	12	4	5	9	21
A1/2	6	5	11	3	6	9	20	Ac	4	4	8	4	5	9	17
Ac	4	4	8	6	4	10	18								
**10.00–10.99**	**10**	**10**	**20**	**10**	**10**	**20**	**40**	**10.00–10.99**	**10**	**10**	**20**	**10**	**10**	**20**	**40**
A1/2	1	1	2	1	1	2	4	A1/2	1	1	2	1	1	2	4
Ac	9	9	18	9	9	18	36	Ac	9	9	18	9	9	18	36
**11.00–11.99**	**10**	**10**	**20**	**10**	**10**	**20**	**40**	**11.00–11.99**	**10**	**10**	**20**	**10**	**10**	**20**	**40**
A1/2		1	1				1	A1/2		1	1				1
Ac	10	9	19	10	10	20	39	Ac	10	9	19	10	10	20	39
**12.00–12.99 to 23.00–23.99**	**120**	**120**	**240**	**120**	**120**	**240**	**480**	**12.00–12.99 to 23.00–23.99**	**120**	**120**	**240**	**120**	**120**	**240**	**480**
Ac	120	120	240	120	120	240	480	Ac	120	120	240	120	120	240	480

Key: Ci—initial cusp formation; Cco—coalescence of cusp; Coc—cusp outline complete; Cr1/2—crown half completed; Cr3/4—crown three-quarters completed; Crc—crown completed; Ri—initial root formation; R1/4—root length less than crown length; R1/2—root length equals crown length; R3/4—three-quarters of root length developed; Rc—root length completed; A1/2—apex closed with wide periodontal ligament width; Ac—apex closed with normal periodontal ligament width.

**Table A7:** Frequencies of the tooth development stages of the second molar for the SA Black and Indian population group.

Maxillary Second Molar (M2)								Mandibular Second Molar (M2)							
Age Range	Female			Male			Grand Total	Age Range	Female			Male			Grand Total
Tooth Stages	Black	Indian	Total	Black	Indian	Total		Tooth Stages	Black	Indian	Total	Black	Indian	Total	
**5.00–5.99**	**10**	**10**	**20**	**10**	**10**	**20**	**40**	**5.00–5.99**	**10**	**10**	**20**	**10**	**10**	**20**	**40**
Cco					1	1	1								
Coc				2	2	4	4	Coc		1	1	1	5	6	7
Cr1/2	6	6	12	4	5	9	21	Cr1/2	5	4	9	5	2	7	16
Cr3/4	1		1	1	2	3	4	Cr3/4	2	2	4	1	2	3	7
Crc	3	4	7	3		3	10	Crc	3	3	6	3	1	4	10
**6.00–6.99**	**10**	**10**	**20**	**10**	**10**	**20**	**40**	**6.00–6.99**	**10**	**10**	**20**	**10**	**10**	**20**	**40**
Coc	1		1				1	Ci		1	1				1
Cr1/2	2	2	4	3	6	9	13	Cr1/2	2	1	3	3	2	5	8
Cr3/4	4	1	5	1		1	6	Cr3/4	2	1	3	1	1	2	5
Crc	3	7	10	6	4	10	20	Crc	6	7	13	6	7	13	26
**7.00–7.99**	**10**	**10**	**20**	**10**	**10**	**20**	**40**	**7.00–7.99**	**10**	**10**	**20**	**10**	**10**	**20**	**40**
Cr1/2	1		1				1	Cr1/2	1		1				1
Cr3/4		1	1	1	1	2	3	Cr3/4		1	1				1
Crc	8	4	12	6	8	14	26	Crc	5	5	10	5	8	13	23
Ri		3	3		1	1	4	Ri	3	1	4	1	1	2	6
R1/4		2	2	1		1	3	R1/4		3	3	2	1	3	6
R1/2	1		1	1		1	2	R1/2				1		1	1
R3/4				1		1	1	R3/4	1		1	1		1	2
**8.00–8.99**	**10**	**10**	**20**	**10**	**10**	**20**	**40**	**8.00–8.99**	**10**	**10**	**20**	**10**	**10**	**20**	**40**
Cr1/2		1	1				1								
Cr3/4					1	1	1								
Crc	1	3	4	4	8	12	16	Crc		3	3	1	3	4	7
Ri	6	3	9	2		2	11	Ri	3		3	1		1	4
R1/4	3	2	5	3	1	4	9	R1/4	5	4	9	5	5	10	19
R1/2		1	1				1	R1/2	2	2	4	3	2	5	9
R3/4				1		1	1	R3/4		1	1				1
**9.00–9.99**	**10**	**10**	**20**	**10**	**10**	**20**	**40**	**9.00–9.99**	**10**	**10**	**20**	**10**	**10**	**20**	**40**
Crc	1	2	3	1	4	5	8	Crc	1	1	2		2	2	4
Ri	1		1				1	Ri				1		1	1
R1/4	4	3	7	4	1	5	12	R1/4	2	3	5	2	3	5	10
R1/2	2	3	5	3	4	7	12	R1/2	6	4	10	6	3	9	19
R3/4	2	2	4	2	1	3	7	R3/4	1	2	3	1	2	3	6
**10.00–10.99**	**10**	**10**	**20**	**10**	**10**	**20**	**40**	**10.00–10.99**	**10**	**10**	**20**	**10**	**10**	**20**	**40**
Crc		1	1				1	Crc		1	1				1
Ri					1	1	1								
R1/4	1	1	2	3	4	7	9	R1/4				1	1	2	2
R1/2	3	2	5	3	4	7	12	R1/2	3	3	6	5	7	12	18
R3/4	2	6	8	3		3	11	R3/4	4	6	10	4	1	5	15
Rc	4		4	1	1	2	6	Rc	3		3		1	1	4
**11.00–11.99**	**10**	**10**	**20**	**10**	**10**	**20**	**40**	**11.00–11.99**	**10**	**10**	**20**	**10**	**10**	**20**	**40**
R1/4		1	1				1								
R1/2				3	2	5	5	R1/2				1	1	2	2
R3/4		1	1	2	5	7	8	R3/4	2	3	5	4	6	10	15
Rc	10	8	18	5	3	8	26	Rc	8	7	15	5	3	8	23
**12.00–12.99**	**10**	**10**	**20**	**10**	**10**	**20**	**40**	**12.00–12.99**	**10**	**10**	**20**	**10**	**10**	**20**	**40**
								R1/2		1	1				1
R3/4		3	3	1		1	4	R3/4	1	1	2	2	2	4	6
Rc	9	7	16	9	10	19	35	Rc	7	7	14	6	8	14	28
A1/2	1		1				1	A1/2	2	1	3	2		2	5
**13.00–13.99**	**10**	**10**	**20**	**10**	**10**	**20**	**40**	**13.00–13.99**	**10**	**10**	**20**	**10**	**10**	**20**	**40**
R3/4		1	1				1								
Rc	9	6	15	5	9	14	29	Rc	1	2	3	7	3	10	13
A1/2	1	3	4	3	1	4	8	A1/2	9	8	17	2	7	9	26
Ac				2		2	2	Ac				1		1	1
**14.00–14.99**	**10**	**10**	**20**	**10**	**10**	**20**	**40**	**14.00–14.99**	**10**	**10**	**20**	**10**	**10**	**20**	**40**
Rc	3	4	7	4	5	9	16	Rc	2	3	5		2	2	7
A1/2	5	5	10	6	3	9	19	A1/2	7	5	12	10	7	17	29
Ac	2	1	3		2	2	5	Ac	1	2	3		1	1	4
**15.00–15.99**	**10**	**10**	**20**	**10**	**10**	**20**	**40**	**15.00–15.99**	**10**	**10**	**20**	**10**	**10**	**20**	**40**
Rc		1	1				1	Rc	1		1				1
A1/2	5	4	9	4	5	9	18	A1/2	6	4	10	4	5	9	19
Ac	5	5	10	6	5	11	21	Ac	3	6	9	6	5	11	20
**16.00–16.99**	**10**	**10**	**20**	**10**	**10**	**20**	**40**	**16.00–16.99**	**10**	**10**	**20**	**10**	**10**	**20**	**40**
A1/2	3	1	4	3	1	4	8	A1/2	4	2	6	3	1	4	10
Ac	7	9	16	7	9	16	32	Ac	6	8	14	7	9	16	30
**17.00–17.99**	**10**	**10**	**20**	**10**	**10**	**20**	**40**	**17.00–17.99**	**10**	**10**	**20**	**10**	**10**	**20**	**40**
Rc	1		1				1	Rc	1		1				1
A1/2	1	1	2	1		1	3	A1/2	2	1	3				3
Ac	8	9	17	9	10	19	36	Ac	7	9	16	10	10	20	36
**18.00–18.99 to 23.00–23.99**	**60**	**60**	**120**	**60**	**60**	**120**	**240**	**18.00–18.99 to 23.00–23.99**	**60**	**60**	**120**	**60**	**60**	**120**	**240**
Ac	60	60	120	60	60	120	240	Ac	60	60	120	60	60	120	240

Key: Ci—initial cusp formation; Cco—coalescence of cusp; Coc—cusp outline complete; Cr1/2—crown half completed; Cr3/4—crown three-quarters completed; Crc—crown completed; Ri—initial root formation; R1/4—root length less than crown length; R1/2—root length equals crown length; R3/4—three-quarters of root length developed; Rc—root length completed; A1/2—apex closed with wide periodontal ligament width; Ac—apex closed with normal periodontal ligament width.

**Table A8:** Frequencies of the tooth development stages of the third molar for the SA Black and Indian population group.

Maxillary Third Molar (M3)								Mandibular Third Molar (M3)							
Age Range	Female			Male			Grand Total	Age Range	Female			Male			Grand Total
Tooth Stages	Black	Indian	Total	Black	Indian	Total		Tooth Stages	Black	Indian	Total	Black	Indian	Total	
**5.00–5.99**	**10**	**10**	**20**	**10**	**10**	**20**	**40**	**5.00–5.99**	**10**	**10**	**20**	**10**	**10**	**20**	**40**
Undeveloped	10	10	20	10	10	20	40	Undeveloped	10	10	20	10	10	20	40
**6.00–6.99**	**10**	**10**	**20**	**10**	**10**	**20**	**40**	**6.00–6.99**	**10**	**10**	**20**	**10**	**10**	**20**	**40**
								Ci	1		1				1
Undeveloped	10	10	20	10	10	20	40	Undeveloped	9	10	19	10	10	20	39
**7.00–7.99**	**10**	**10**	**20**	**10**	**10**	**20**	**40**	**7.00–7.99**	**10**	**10**	**20**	**10**	**10**	**20**	**40**
Ci		2	2	2		2	4	Ci	1	2	3	2		2	5
Cco	1		1	1		1	2	Cco				1		1	1
Coc				1		1	1	Cr1/2				2		2	2
Crc				1		1	1								
Undeveloped	9	8	17	5	10	15	32	Undeveloped	9	8	17	5	10	15	32
**8.00–8.99**	**10**	**10**	**20**	**10**	**10**	**20**	**40**	**8.00–8.99**	**10**	**10**	**20**	**10**	**10**	**20**	**40**
Ci	1	3	4	2	3	5	9	Ci	6	3	9	4	3	7	16
Cco	7		7	1		1	8	Cco	4		4	2		2	6
Coc	2	1	3	4		4	7	Coc		2	2				2
Cr1/2				1		1	1	Crc				1		1	1
Undeveloped		6	6	2	7	9	15	Undeveloped		5	5	3	7	10	15
**9.00–9.99**	**10**	**10**	**20**	**10**	**10**	**20**	**40**	**9.00–9.99**	**10**	**10**	**20**	**10**	**10**	**20**	**40**
Ci		4	4		3	3	7	Ci	1	5	6	4	5	9	15
Cco	4		4	3	2	5	9	Cco	3	1	4	2	1	3	7
Coc	1	1	2	2	1	3	5	Coc	1		1	2	1	3	4
Cr1/2	3	1	4	4	2	6	10	Cr1/2	1		1		2	2	3
Crc	1		1	1		1	2	Cr3/4	2		2				2
R1/2		1	1				1	Crc	1		1	1		1	2
Undeveloped	1	3	4		2	2	6	R1/2		1	1				1
								Undeveloped	1	3	4	1	1	2	6
**10.00–10.99**	**10**	**10**	**20**	**10**	**10**	**20**	**40**	**10.00–10.99**	**10**	**10**	**20**	**10**	**10**	**20**	**40**
Ci	1	1	2				2	Ci		2	2		2	2	4
Cco		1	1	2	1	3	4	Cco	1	1	2	1	3	4	6
Coc		2	2		5	5	7	Coc	2		2		2	2	4
Cr1/2	4	3	7	4		4	11	Cr1/2	3	4	7	4		3	10
Cr3/4	2	2	4	2		2	6	Cr3/4	1	1	2	2	1	3	5
Crc	3		3	2	1	3	6	Crc	2		2	3		4	6
Undeveloped		1	1		3	3	4	R1/4	1		1				1
								Undeveloped		2	2		2	2	4
**11.00–11.99**	**10**	**10**	**20**	**10**	**10**	**20**	**40**	**11.00–11.99**	**10**	**10**	**20**	**10**	**10**	**20**	**40**
Ci					2	2	2	Ci		1	1		2	2	3
Cco				1		1	1	Cco				1		1	1
Coc		1	1	1	2	3	4	Coc		1	1	1	3	4	5
Cr1/2	1	3	4	2	3	5	9	Cr1/2	1	2	3	1	1	2	5
Cr3/4	4	3	7	4	1	5	12	Cr3/4	4	4	8	4	1	5	13
Crc	3	1	4	2	2	4	8	Crc	3	1	4	3	2	5	9
Ri	1		1				1	Ri	1		1				1
R1/4	1		1				1	R1/4	1		1				1
Undeveloped		2	2				2	Undeveloped		1	1		1	1	2
**12.00–12.99**	**10**	**10**	**20**	**10**	**10**	**20**	**40**	**12.00–12.99**	**10**	**10**	**20**	**10**	**10**	**20**	**40**
Coc		1	1				1	Ci		1	1				1
Cr1/2	1	2	3		1	1	4	Coc		1	1	1	1	2	3
Cr3/4	1		1	2	1	3	4	Cr1/2		2	2	1	1	2	4
Crc	5	7	12	8	8	16	28	Cr3/4	2	1	3				3
Ri	1		1				1	Crc	5	4	9	7	7	14	23
R1/2	2		2				2	Ri				1		1	1
								R1/4	1	1	2		1	1	3
								R1/2	2		2				2
**13.00–13.99**	**10**	**10**	**20**	**10**	**10**	**20**	**40**	**13.00–13.99**	**10**	**10**	**20**	**10**	**10**	**20**	**40**
Ci		1	1				1	Ci		1	1				1
Cr1/2		2	2	1	1	2	4	Cr1/2	1	2	3	2		2	5
Cr3/4					2	2	2	Cr3/4					2	2	2
Crc	3	6	9	6	5	11	20	Crc	1	5	6	4	2	6	12
Ri	1		1	2		2	3	Ri	1		1	2	4	6	7
R1/4	1	1	2	1	1	2	4	R1/4	3		3	2	2	4	7
R1/2	2		2				2	R1/2	2	2	4				4
R3/4	1		1				1	R3/4	1		1				1
Rc	2		2				2	Rc	1		1				1
Undeveloped					1	1	1	Undeveloped							
**14.00–14.99**	**10**	**10**	**20**	**10**	**10**	**20**	**40**	**14.00–14.99**	**10**	**10**	**20**	**10**	**10**	**20**	**40**
Cr3/4		1	1				1	Cr3/4	1	2	3				3
Crc	2		2	2	1	3	5	Crc	2	4	6	2	1	3	9
Ri	3	5	8	1	4	5	13	R1/4	1	1	2	3	1	4	6
R1/4	1	1	2	3		3	5	R1/2	4		4	2	6	8	12
R1/2	1	1	2	1	3	4	6	R3/4	2	2	4	2	2	4	8
R3/4	2	1	3	2	2	4	7	Rc		1	1	1		1	2
Rc		1	1	1		1	2								
Undeveloped	1		1				1								
**15.00–15.99**	**10**	**10**	**20**	**10**	**10**	**20**	**40**	**15.00–15.99**	**10**	**10**	**20**	**10**	**10**	**20**	**40**
Crc	1	2	3	2		2	5	Crc	1	1	2	2	1	3	5
Ri		1	1	1	2	3	4	R1/4	1	4	5	1		1	6
R1/4	3	3	6	3	3	6	12	R1/2	4	2	6	4	4	8	14
R1/2	2	2	4	1	1	2	6	R3/4		2	2	1	3	4	6
R3/4	1	1	2	1	2	3	5	Rc	3	1	4	2	2	4	8
Rc	2	1	3	2	2	4	7	A1/2	1		1				1
A1/2	1		1				1								
**16.00–16.99**	**10**	**10**	**20**	**10**	**10**	**20**	**40**	**16.00–16.99**	**10**	**10**	**20**	**10**	**10**	**20**	**40**
Crc		2	2				2	Cr1/2		1	1				1
Ri		2	2				2	Crc		1	1				1
R1/4	4	1	5		1	1	6	R1/4		1	1				1
R1/2	1	4	5	1	4	5	10	R1/2	3	6	9	3	3	6	15
R3/4	3	1	4	5	2	7	11	R3/4	4	1	5	4	4	8	13
Rc	2		2	4	3	7	9	Rc	3		3	3	2	5	8
								A1/2					1	1	1
**17.00–17.99**	**10**	**10**	**20**	**10**	**10**	**20**	**40**	**17.00–17.99**	**10**	**10**	**20**	**10**	**10**	**20**	**40**
R1/4	2	1	3	2		2	5	R1/4	1	1	2				2
R1/2	1	1	2	2		2	4	R1/2	2	2	4	2		2	6
R3/4	3	4	7	1	5	6	13	R3/4	3	4	7	4	5	9	16
Rc	2	3	5	4	4	8	13	Rc	2	3	5	3	4	7	12
A1/2	1		1				1	A1/2	2		2				2
Ac	1	1	2	1	1	2	4	Ac				1	1	2	2
**18.00–18.99**	**10**	**10**	**20**	**10**	**10**	**20**	**40**	**18.00–18.99**	**10**	**10**	**20**	**10**	**10**	**20**	**40**
Crc		1	1				1	R1/2		3	3				3
R1/2		2	2				2	R3/4		1	1		1	1	2
R3/4		4	4				4	Rc	5	5	10	5	5	10	20
Rc	5	2	7	2	5	7	14	A1/2	3	1	4	1	1	2	6
A1/2	3	1	4	5	2	7	11	Ac	2		2	4	3	7	9
Ac	2		2	3	3	6	8								
**19.00–19.99**	**10**	**10**	**20**	**10**	**10**	**20**	**40**	**19.00–19.99**	**10**	**10**	**20**	**10**	**10**	**20**	**40**
Rc	2	5	7	2	5	7	14	R3/4					1	1	1
A1/2	5	2	7	5	1	6	13	Rc	3	5	8	3	5	8	16
Ac	3	3	6	3	4	7	13	A1/2	4	3	7	4	1	5	12
								Ac	3	2	5	3	3	6	11
**20.00–20.99**	**10**	**10**	**20**	**10**	**10**	**20**	**40**	**20.00–20.99**	**10**	**10**	**20**	**10**	**10**	**20**	**40**
R3/4		1	1				1	Rc	1	2	3	2	1	3	6
Rc		2	2	1	1	2	4	A1/2		5	5	1	5	6	11
A1/2	1	4	5	1	5	6	11	Ac	9	3	12	7	4	11	23
Ac	9	3	12	8	4	12	24								
**21.00–21.99**	**10**	**10**	**20**	**10**	**10**	**20**	**40**	**21.00–21.99**	**10**	**10**	**20**	**10**	**10**	**20**	**40**
Rc					1	1	1	Rc		1	1		2	2	3
A1/2		1	1		1	1	2	A1/2		1	1		2	2	3
Ac	10	9	19	10	8	18	37	Ac	10	8	18	10	6	16	34
**22.00–22.99 to 23.00–23.99**	**20**	**20**	**40**	**20**	**20**	**40**	**80**	**22.00–22.99 to 23.00–23.99**	**20**	**20**	**40**	**20**	**20**	**40**	**80**
Ac	20	20	40	20	20	40	80	Ac	20	20	40	20	20	40	80

Key: Ci—initial cusp formation; Cco—coalescence of cusp; Coc—cusp outline complete; Cr1/2—crown half completed; Cr3/4—crown three-quarters completed; Crc—crown completed; Ri—initial root formation; R1/4—root length less than crown length; R1/2—root length equals crown length; R3/4—three-quarters of root length developed; Rc—root length completed; A1/2—apex closed with wide periodontal ligament width; Ac—apex closed with normal periodontal ligament width.
